# The Role of miR-378a in Metabolism, Angiogenesis, and Muscle Biology

**DOI:** 10.1155/2015/281756

**Published:** 2015-12-29

**Authors:** Bart Krist, Urszula Florczyk, Katarzyna Pietraszek-Gremplewicz, Alicja Józkowicz, Jozef Dulak

**Affiliations:** Department of Medical Biotechnology, Faculty of Biochemistry, Biophysics and Biotechnology, Jagiellonian University, Gronostajowa 7, 30–387 Krakow, Poland

## Abstract

MicroRNA-378a (miR-378a, previously known as miR-378) is one of the small noncoding RNA molecules able to regulate gene expression at posttranscriptional level. Its two mature strands, miR-378a-3p and miR-378a-5p, originate from the first intron of the peroxisome proliferator-activated receptor gamma, coactivator 1 beta (*ppargc1b*) gene encoding PGC-1*β*. Embedding in the sequence of this transcriptional regulator of oxidative energy metabolism implies involvement of miR-378a in metabolic pathways, mitochondrial energy homeostasis, and related biological processes such as muscle development, differentiation, and regeneration. On the other hand, modulating the expression of proangiogenic factors such as vascular endothelial growth factor, angiopoietin-1, or interleukin-8, influencing inflammatory reaction, and affecting tumor suppressors, such as SuFu and Fus-1, miR-378a is considered as a part of an angiogenic network in tumors. In the latter, miR-378a can evoke broader actions by enhancing cell survival, reducing apoptosis, and promoting cell migration and invasion. This review describes the current knowledge on miR-378a linking oxidative/lipid metabolism, muscle biology, and blood vessel formation.

## 1. Introduction

Cell metabolism governing the growth and functioning of each cell and a whole organism refers to chemical transformations and enzyme-catalyzed energy producing and energy utilizing reactions of carbohydrates, proteins, and lipids. Amongst the most metabolically active organs are liver, brain, gut, kidneys, and heart [1–3]. Although the rate of metabolic reactions is lower in skeletal muscles, they account for around 20% of the total energy expenditure due to a 50–60% contribution to a total body mass [3]. Several microRNAs were reported to control processes related to metabolism such as insulin secretion (miR-9, miR-375), adipocyte differentiation (miR-143), fatty acid metabolism (miR-122), and myogenesis (miR-1, miR-133a, miR-133b, and miR-206) (reviewed in [4]). Of potential meaning is also miR-378a, located in the gene encoding master metabolic regulator, peroxisome proliferator-activated receptor gamma, coactivator 1 beta (PGC-1*β*) [5]. miR-378a was found to affect lipid and xenobiotic metabolism, lipid storage, mitochondrial function, and shift towards a glycolytic pathway (Warburg effect) [5, 6]. Moreover, it affects muscle differentiation via regulation of myogenic repressor, MyoR [7]. Because nutrients supply for metabolic processes is a matter of circulation, metabolically active tissues require high vascular density. Recently, miR-378a was reported to regulate tumor angiogenesis mainly via inhibition of tumor suppressors SuFu and Fus-1 [8, 9]. Thus, a growing body of evidence suggests a role of miR-378a as a mediator controlling reciprocally dependent processes such as metabolism, muscle differentiation/regeneration, and angiogenesis.

## 2. MicroRNAs

MicroRNAs (miRNAs; miRs) are small noncoding RNA molecules with an average length of 21-22 nucleotides which can regulate gene expression posttranscriptionally by targeting mostly the 3′untranslated region (3′UTR) of mRNAs. However, miRNA target sites were also found on the 5′UTR regions of human mRNA [10]. Since their discovery in* C. elegans *in 1993 [11], miRNAs currently can be recognized as potent players in wide spectrum of biological processes like development, differentiation, cellular defense mechanisms, and others. Conservative estimates state that over 30% of mRNA expression is regulated by miRNAs [12, 13]. However, others suggest that even up to 60% of the mRNA expression is targeted by miRNAs [14]. miRNAs are often located in the introns of coding genes or noncoding sequences but can also be located in exons. Intronic miRNAs can be expressed together with their host gene mRNA being derived from a common RNA transcript [15, 16]. Other miRNAs can also have their own promoters, which enable independent expression, or can be organized in clusters sharing a common transcriptional regulation [17, 18].

**Figure 1:**
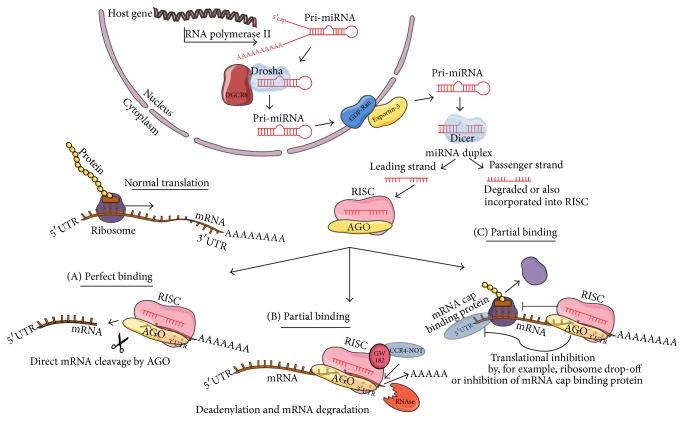
miRNA biogenesis. miRNAs are transcribed as mRNA transcripts from the genome by polymerase II as pre-miRs. Endoribonuclease drosha and DGCR8 excise pre-miRs from the primary transcripts. Pri-miRs are exported from the nucleus by exportin-5. An endoribonuclease dicer processes the pre-miRNA and removes the hair loop sequence, creating a double stranded miRNA duplex. One or both strands can be incorporated in RNA-induced silencing complex RISC, which allows the miRNA to suppress translation of their target mRNA or cleave the mRNA and lead to the degradation of it. miRNA-induced RISC can act on their targets by three ways. When there is perfect pairing between the miRNA sequence and its target site, the mRNA is cleaved (A). If the pairing is partial, deadenylation of the mRNA via recruitment of the CCR4-NOT complex takes place and the poly-A tail is lost, leaving the mRNA vulnerable to RNAse activity and mRNA degradation (B). As a second manner of action when pairing is not perfect, the miRNA-induced RISC can also induce repression of translation by blocking initiation or further steps of translation, by mechanisms such as, for example, the promotion of ribosome drop-off from the mRNA transcript or destabilization of the mRNA cap binding protein (C).

miRNAs transcription is RNA polymerase II-dependent [17]. In the case of miRNAs that are encoded in their own genes, the primary miRNA transcript (pri-miRNA) is several kilobases long, while miRNAs encoded in intronic regions of other genes (miRtrons) have shorter transcripts. The miRNA stem loop is excised from pri-miRNA by endoribonuclease drosha/DGCR8 (microprocessor complex) and a hairpin called pre-miRNA is exported from the nucleus by exportin-5 in a Ran-GTP dependent manner [19]. An endoribonuclease dicer removes the hairpin loop sequence from pre-miRNA, creating a double stranded miRNA duplex. Depending on the relative stability of the miRNA duplex, one or, more rarely, both strands can be incorporated in a multiprotein RNA-induced silencing complex (RISC). When there is perfect pairing between the miRNA sequence and its target site, mRNA is cleaved by a protein part of the RISC called argonaute (AGO). If the pairing is partial, deadenylation of the mRNA via recruitment of the CCR4-NOT complex by the GW182 proteins inside the RISC takes place and the poly-A tail is lost, leaving the mRNA vulnerable to RNase activity, ubiquitination, and mRNA degradation. Alternatively, miRNA-induced RISC can also cause repression of translation by mechanisms such as, for example, the promotion of ribosome drop-off from the mRNA transcript or destabilization of the mRNA binding cap protein (Figure 1) (reviewed in [20, 21]).

## 3. miR-378a: Basics

miR-378a is embedded in the first intron of the* ppargc1b *gene encoding PGC-1*β* [5]. The pre-miR gives rise to a leading strand (miR-378a-3p, previous IDs for murine sequence: mmu-miR-422b, mmu-miR-378, and mmu-miR-378-3p; for human: hsa-miR-422b and hsa-miR-378) and a passenger strand (miR-378a-5p, previous IDs for murine sequence: mmu-miR-378, mmu-miR-378^*∗*^, and mmu-miR-378-5p; for human: hsa-miR-378 and hsa-miR-378^*∗*^). miRNA-378a-3p mature strand was first identified in 2004 in humans (originally named miR-422b) [22]. Recently, other miRs with similar sequences but other localizations in the genome have been discovered and named: mmu-miR-378b,c,d in mouse and hsa-miR-378-b,c,d1,d2,e,f,g,h,i,j in human [23–27] (Table 1). In humans, miR-378a is by far the most expressed of the miR-378 sequences, with 7030 reads per million, in 78 experiments during deep sequencing, compared with 101–3220 reads per million, in 42–72 experiments for the other forms, respectively. In mice, miR-378a and miR-378b have similar expression levels, at 11700 and 11000 reads per million (miRBase, version 21, September 2015) [28]. The sequence of miR-378a mature strands is highly conserved between species, with the miR-378a-5p strand being identical in both human and mice and the miR-378a-3p strand only differing in one nucleotide (Table 2) [6, 27].

PGC-1*β* may regulate several facets of energy metabolism such as mitochondrial biogenesis, thermogenesis, and glucose and fatty acid metabolism [6]. Both strands of miR-378a are coexpressed with PGC-1*β* as shown, for example, in the liver and during adipocyte differentiation [6, 29]. The coexpression of miR-378a with its host gene implies they may share the same transcriptional activators, and miR-378a might be involved in similar processes as PGC-1*β*. Accordingly, high levels of (porcine) miR-378-1 (Table 2) expression are found in developing muscle, postnatal muscle, and myocardium and in brown adipose tissue [29, 30].

To date, only a limited number of miR-378a targets, which can be predicted based on* in silico* analysis, have been experimentally validated. The latter, however, imply a role of miR-378a in mitochondrial energy homeostasis, glycolysis, and skeletal muscle development and in tumor angiogenesis and other processes (Table 3).

## 4. miR-378a in Metabolism

A major source of energy production comprises oxidation of glucose in glycolysis followed by oxidation of pyruvate in well-oxygenated cells (or followed by lactic acid fermentation in cancer, the Warburg effect) and from *β*-oxidation of lipids, which yields even more ATP per gram then carbohydrates metabolism. A complicated net of metabolic pathways requires advanced regulation by signaling molecules and hormones.

A location of miR-378a in the gene encoding PGC-1*β* [5] implies an involvement of miR-378a in metabolic pathways. Unlike its homologue, PGC-1*α*, the expression of PGC-1*β* is not elevated in response to cold exposure [31] but occurs in response to hypoxia, exercise, caloric restriction, or aging (reviewed in [32]). PGC-1*β* is preferentially expressed in tissues with relatively high mitochondrial content, such as heart, skeletal muscle, and brown adipose tissue [6]. In 2002, PGC-1*β* was first cloned and shown to be upregulated in the liver during fasting [31]. PGC-1*β* strongly activates hepatic nuclear factor 4 (HNF4) and PPAR*α*, both of these nuclear receptors being important for the adaptation of hepatocytes to the effects of fasting. These findings could hint to a possible role of PGC-1*β* in the regulation of gluconeogenesis and fatty acid oxidation in the liver [31]. PGC-1*β* is also involved in the regulation of energy expenditure or in the pathway of estrogen receptor-related receptors (ERRs) [33–37]. Since miRNAs originating in the introns of host genes may modulate the protein encoded by their parental genes and may be involved in the same mechanisms [38–40], miR-378a is proposed to be involved in the metabolic pathways affected by PGC-1*β* [6].

It was reported that mice lacking the first intron of the* ppargc1b* gene (and thus miR-378a) have a significantly higher oxygen capacity and mitochondrial function [6]. Such mice also exhibit a resistance to high fat induced obesity. They identified a mediator complex subunit 13 (MED13), involved in nuclear receptor signaling, and carnitine acetyltransferase (CRAT), a mitochondrial enzyme involved in fatty acid metabolism, as targets of miR-378a-5p and miR-378a-3p, respectively [6]. It implies that miR-378a plays a regulatory role in lipid metabolism. miR-378a-5p regulated also cytochrome P450 2E1 (CYP2E1) being involved in the metabolism of, for example, drugs and toxins [41].

In addition, it has been discovered that transcription factor nuclear respiratory factor-1 (NRF-1), a critical regulator of the expression of some important metabolic genes in mitochondria regulating cellular growth, is inhibited by miR-378a-3p [42]. Thus, miR-378a can be considered as a regulator of mitochondrial function in cells overexpressing miR-378a.

Moreover, miR-378a-5p inhibits the mRNAs of ERR*γ* and GA-binding protein-*α* in breast cancer, which both interact with PGC-1*β* and together control oxidative metabolism [5]. This leads to a reduction of tricarboxylic acid gene expression and oxygen consumption and an increase in lactate production, which shifts cells from an oxidative towards a glycolytic pathway. In this way, miR-378a-5p is believed to be a switch regulating the Warburg effect in breast cancer [5]. Moreover, in situ hybridization experiments in this study showed that miR-378a-5p expression correlates with progression of breast cancer [5]. The proposed regulating role of miR-378a-5p on the Warburg effect is in parallel with the effects of PGC-1*β*, which mediates gluconeogenesis and fatty acid metabolism after periods of fasting or intense exercise [31]. Coactivation by PGC-1*β* of ERR*α* and PPAR*α* makes muscle fibers in PGC-1*β* transgenic mice more rich in mitochondria and highly oxidative [43]. Accordingly, such animals were able to run for longer times and at higher workloads [43].

Increased glycolytic rates and increased cell proliferation can be related to lactate production by lactate dehydrogenase (LDH). LDHA was found to be a direct target of miR-378a in the study of Mallat et al. [44]. In this way, hsa-miR-378a-3p represses cell growth and increases cell death by targeting LDHA. Of note, hsa-miR-378a-3p and hsa-miR-378a-5p had opposite effects on LDHA expression. LDHA was significantly downregulated by miR-378a-3p overexpression and upregulated by miR-378a-5p overexpression [44].

In addition, miR-378a is also considered as an important factor in adipogenesis and lipid storage. There is a complex family of factors regulating those processes such as insulin [45], insulin-like growth factors (IGFs), glucagon, and thyroid hormones T3 and T4 (reviewed in [46–49]). As mentioned before, it was demonstrated that miR-378a-knockout mice do not get fat after 8 weeks of high fat diet [6]. Such animals show an enhanced mitochondrial fatty acid metabolism and have elevated oxidative capacity of tissues targeted by insulin (e.g., liver, muscles, and adipose tissues) [6]. In accordance with that, it was shown that mature strands of bta-miR-378-1 (Table 1) are expressed at higher level in cows with high (versus low) amount of back fat [50]. Similarly, an inhibition of both mmu-miR-378a-3p and its host gene, PGC-1*β*, by the flavonoid fisetin lowered the accumulation of fat in the liver [42]. Interestingly, mmu-miR-378a-5p was downregulated in mice that were fed a high fat diet for five months [51]. In addition, miR-378a is highly induced during adipogenesis [29]. Overexpression of miR-378a-3p/-5p during adipogenesis increased the transcriptional activity of CCAAT/enhancer-binding proteins (cEBP) alpha and beta, which can stimulate the expression of leptin, a hormone produced mainly by adipocytes which controls the homeostasis of body weight [29] (reviewed in [52, 53]). On the other hand, TNF-*α*, IL-6, and leptin are reported to increase the expression of miR-378a-3p in mature human adipocytes* in vitro* [54]. These cytokines are mainly secreted in the adipose tissue and are suggested to be involved in development of insulin resistance [55, 56]. In addition, miR-378a-3p was shown to target insulin growth factor 1 receptor (IGF1R) and reduce the Akt signaling cascade in cardiomyocytes during cardiac development [57]. Moreover, in tissues where IGF1 levels were high (e.g., fibroblasts and fetal hearts), miR-378-3p levels were very low, showing an inverse relation and suggesting a negative feedback loop between miR-378a-3p, IGF1R, and IGF1 [57].

As already mentioned, PGC-1*β* is a coactivator of PPAR*γ* [5]. The latter functions as a master regulator of adipogenesis and is involved in the formation of peroxisomes and the catabolism of very long chain fatty acids [58, 59]. PPAR*γ* facilitates also the storage of fat in part by inhibiting leptin [60]. Accordingly, the amount of adipose tissue does not increase in mice lacking PPAR*γ* when they are fed a high fat diet [61]. It was also reported that in cultured adipocytes mmu-miR-378a and PGC-1*β* expression is PPAR*γ*, or rosiglitazone (a PPAR*γ* ligand), dependent, finding two peroxisome proliferator response elements in the miR-378a loci [62]. On the other hand, overexpression of miR-378a elevated the expression of PPAR*γ* isoform 2 [29], suggesting positive feedback loop and confirming the involvement of miR-378a in the storage of fat.

There are several activators known to induce expression of PPAR*γ* such as the members of the E2F transcription factor family and prostaglandin J-2 (PGJ-2) [63–65]. The latter may act through RAR-related orphan receptor alpha (RORA), which is frequently found in myocardium [66]. In addition to PPAR*γ*, RORA regulates also MyoD, a major transcription factor involved in skeletal muscle differentiation [67, 68]. Interestingly, RORA is a possible (but not yet validated) target for miR-378a-3p [69].

A proteomics-based study revealed several other proteins that are potentially targeted by rat miR-378a-3p or miR-378a-5p. miR-378a-3p was shown to regulate mannose-1-phosphate guanylyltransferase (GDP), dimethylarginine dimethylaminohydrolase 1 (DDAH1), and lactate dehydrogenase A (LDHA); all those proteins are participating in metabolic processes [44]. On the other hand, tropomyosin beta chain, which is involved in the regulation of ATPase activity, was found to be a target of miR-378a-5p [44].

## 5. miR-378a in Muscle Development, Differentiation, and Regeneration

High levels of murine and rat miR-378a-3p, miR-378a-5p, and porcine miR-378-1 are reported in both developing and adult skeletal muscles [7, 30, 44]. miR-378a expression is enhanced during skeletal muscle differentiation [30].

MyoD and MyoG play a role in the processes of myogenesis and muscle regeneration, in which dormant satellite cells are activated upon muscle damage and start proliferating and differentiating into muscle fibers (reviewed in [70, 71]). It has been shown that miR-378a-3p targets the myogenic repressor MyoR during myoblast differentiation, which directly inhibits MyoD [7]. On the other hand, MyoD is upregulated in response to miR-378a-3p overexpression and, conversely, the level of miR-378a-3p may be enhanced by MyoD [7]. Thus, there is evidence for a feedback loop in which miR-378a-3p regulates muscle differentiation via inhibiting MyoR, leading to an increase of MyoD, which in turn enhances miR-378a-3p [7].

It has been suggested by Davidsen et al. that miR-378a may also control the development of skeletal muscle mass after training [72]. In this study, miR-378a (strand not specified) was significantly downregulated in men who obtained low training-induced muscle mass gain compared to men who obtained high training-induced muscle mass gain [72].

A growing body of data shows a role of miR-378a-3p in the myocardium. miR-378a-3p is expressed mostly by cardiomyocytes, but not by nonmuscle cells, whereas the level of miR-378a-5p was reported to be very low in the heart [57]. Fang et al. showed that miR-378-3p is significantly downregulated both* in vitro* in cardiomyocytes cell cultures exposed to hypoxia and* in vivo* during myocardial injury in rats [73]. Overexpression of miR-378a-3p enhanced cell viability and inhibited apoptosis via caspase-3 inhibition [73]. In contrast to this finding, another study found that miR-378a-3p downregulation enhanced the survival of cardiac stem cells via focal adhesion kinase activation and releasing connective tissue growth factor (CTGF), the latter being a target of miR-378a-3p [74]. miR-378a inhibition enhanced cardiomyocytes survival after H_2_O_2_ treatment [57]. Overexpression of miR-378a-3p in the study of Knezevic et al. increased apoptosis of cardiomyocytes via the direct targeting of IGF1R leading to a decrease of Akt signaling [57]. This is in opposition to the previously mentioned study of Fang et al. which showed apoptosis was decreased during miR-378a-3p overexpression due to targeting of caspase-3 [73]. The converse findings of the studies could be explained by different models used by Knezevic et al. and Fang et al. Because of those discrepancies, the role of miR-378a in apoptosis of cardiomyocytes requires further investigation. The finding that miR-378a-3p affects both IGF1R and the Akt pathway was confirmed [75] in a study which found that overexpression of miR-378a-3p in rhabdomyosarcoma suppressed IGF1R expression and affected phosphorylation of the Akt protein [75]. miR-378a-5p was shown to target heat shock protein 70.3 (Hsp70.3) in mouse hearts in normoxic conditions, but in hypoxic conditions a transcript variant of Hsp70.3 without miR-378a-5p target site in its 3′-UTR is not repressed and can exert its cytoprotective properties [76].

Potential involvement of miR-378a in cardiac remodeling was also proposed. miR-378a-3p prevented cardiac hypertrophy by targeting either Ras signaling or the mitogen-activated protein kinase (MAPK) pathway [77, 78].

More studies on the effect of miR-378a expression in muscle disorders would also be desirable. In both Golden Retriever muscular dystrophy dogs and Duchenne muscular dystrophy patients, miR-378a expression was dysregulated, suggesting some relation between miR-378a expression and muscle dystrophy [79].

All in all, these findings suggest miR-378a-3p can be considered as an important player in cardiac development, remodeling, and hypertrophy.

## 6. miR-378a in Angiogenesis

Angiogenesis comprises development of new blood vessels from existing ones, regulated by cytokines and growth factors such as, for example, vascular endothelial growth factor (VEGF), platelet-derived growth factor (PDGF), and angiopoietin-1 (Ang-1). Their expression can be posttranscriptionally controlled by microRNAs such as miR-126, miR-296, miR-210, miR-21, and the miR-17~92 cluster [80] (reviewed in [81]).

Skeletal muscles and heart muscle are tissues which, due to their oxygen and energy consumption, need to be sufficiently vascularized. One of the major regulators of angiogenesis is the hypoxia-inducible factor-1 (HIF-1), which controls over 100 genes [82] involved mainly in the glycolytic pathway and blood vessel formation, including VEGF-A or interleukin-8 [83–85]. VEGF is generally induced by hypoxia, while IL-8 in at least some cancers and endothelial cells can be diminished by HIF-1 via inhibition of c-Myc and Sp-1 transcription factors [86, 87]. c-Myc, known as a regulator of cell cycle progression, apoptosis, and cellular transformation, is also a potent activator of PGC-1*β* and, in turn, miR-378a-3p, upregulating their expression [88].

In addition, miR-378a has been shown to affect VEGF-A in two ways. Human hsa-miR-378a-5p (by the study of Hua et al. named as miR-378) can directly affect VEGF-A by competing with hsa-miR-125a for the same seed-region in the VEGF-A 3′UTR causing upregulation of VEGF-A [89]. miR-378a-5p can also indirectly regulate VEGF-A affecting sonic hedgehog (SHH) signaling via Sufu inhibition, which is an inhibitory component of this signaling pathway [8]. The SHH pathway in turn can upregulate VEGF-A and also other regulators of blood vessels formation, Ang-1 and Ang-2 expression [90–92]. Increased expression of VEGF-A, as well as PDGF*β* and TGF*β*1, was also seen in mesenchymal stromal cells (MSCs) transfected with rno-miR-378a-5p [93].

In skeletal muscles, VEGF-induced angiogenesis appears not to be regulated by the well-known HIF pathway but by PGC-1*α*, which coactivates estrogen-related receptor alpha (ERR-*α*) on binding sites in the promoter and the first intron of the VEGF gene, inducing its expression [94]. This angiogenic pathway shows new roles for PGC-1*α* and ERR-*α*, which are important regulators of mitochondrial activity in response to stimuli like exercise. If there might be a role for PGC-1*β* in this pathway, it is yet to be examined. It is noteworthy, however, that miR-378a-5p is known to affect the estrogen receptors by inhibiting ERR*γ*, another estrogen-related receptor [5].

A role for miR-378a in cell cycle regulation and stimulation of cell growth is also proposed. In human mammary epithelial and breast cancer cell lines, miR-378a-3p can target the antiproliferative protein TOB2, which is a suppressor of cyclin D1, which in turn is required for cell cycle G1-phase to S-phase progression [88]. Enhancing endothelial cell proliferation via cell cycle regulation contributes to the angiogenic process. Whether miR-378 affects endothelial cell proliferation by regulation of cell cycle remains to be established.

The role of miR-378a in the formation of blood vessels nourishing tumor and enabling tumor growth was revealed. miR-378a was found to be differentially regulated in different types of cancers [95] being downregulated in gastric cancer [96, 97], oral [98], and colon carcinoma [99], while being upregulated in renal [100] and lung cancer [9, 101]. Since it is also changed in serum or plasma of patients with prostate cancer [102], renal cancer [100, 103], and gastric cancer [104] and frequently found to be overexpressed in cryopreserved bone marrow mononuclear cells from acute myeloid leukemia patients [105], miR-378a might be considered as a biomarker.

The role of miR-378a in tumorigenesis, tumor growth, and tumor vascularization was revealed for the first time by Lee and coworkers in glioblastoma [8]. They showed that miR-378a-5p enhances cell survival, reduces caspase-3 activity, and promotes tumor growth and angiogenesis, through repression of two tumor suppressors, Sufu and Fus-1 [8]. Strikingly, nude mice injected with miR-378a-5p transfected cancer cells formed tumors of bigger volume and with larger blood vessels compared to GFP-transfected cells. On the other hand, high expression of miR-378a-5p in NSCLC correlated with brain metastases due to higher cell migration, invasion, and tumor angiogenesis [9]. Another study confirmed the downregulation of Fus-1 by miR-378a-5p and showed that in the HepG2 liver cancer cells miR-378a-5p overexpression enhanced proliferation, migration, and, when injected in mice, invasion [106]. Also in rhabdomyosarcoma, enhanced expression of miR-378a, VEGF, and MMP9 correlated with increased vascularization and metastasis [107]. Taken together, these studies suggest that miR-378a may serve as a prognostic marker in cancer due to its effects on angiogenesis.

Our recent data confirmed the proangiogenic effect of miR-378a (both strands) in non-small cell lung carcinoma (NSCLC) and pointed at its correlation with heme-degrading enzyme, heme oxygenase-1 (HO-1). An involvement of HO-1 in angiogenesis and VEGF-A as well as IL-8 signaling was shown by us previously [108]; however, its action in tumors seems to be complex [109]. In NCI-H292 cell line overexpressing HO-1, miR-378a (both strands) levels decreased [101]. Conversely, when HO-1 was silenced using siRNA, miR-378a expression was enhanced. Also overexpression of the miR-378a precursor sequence diminished HO-1 expression. Conditioned medium from NCI-H292 cells overexpressing miR-378a enhanced angiogenic potential of HMEC-1 endothelial cell line. Tumors formed by such cells in subcutaneous xenografts showed enhanced growth, vascularization, oxygenation, and distal metastasis* in vivo *[101]. These interactions between miR-378a and HO-1 were confirmed in our studies on the role of the Nrf-2 transcription factor/HO-1 axis in NSCLC cell lines [110, 111].

On the other hand, enhanced expression of mmu-miR-378a-5p in 4T1 murine breast cancer cells decreased the proliferation, migration, and invasiveness of these cancer cells* in vitro *and* in vivo *by targeting fibronectin, resulting in inhibition of tumor growth [112].

Recent study showed that miR-378a may act as a biomarker for response to antiangiogenic treatment in ovarian cancer [113]. Low expression of miR-378a was associated with longer progressive-free survival in patients with recurrent ovarian cancer treated with the antiangiogenic drug bevacizumab [113]. Overexpression of the miR-378a precursor in ovarian cancer cells altered expression of genes associated with angiogenesis (ALCAM, EHD1, ELK3, and TLN1), apoptosis (RPN2, HIPK3), and cell cycle regulation (SWAP-70, LSM14A, and RDX) [113]. High miR-378a (strand not specified) expression in renal carcinoma correlated with higher levels of endothelial surface marker CD34 in these tumors [114].

Notably, a recent study suggested clinical relevance for miR-378a in metastatic colorectal cancer, in which enhanced miR-378a expression significantly improved the sensitivity to cetuximab treatment in these patients [115].

Interestingly, recent data indicate a role of miR-378a in stem cells. miR-378a-5p transfection of MSCs has been shown to enhance their survival and angiogenic potential under hypoxic conditions* in vitro *[93]. In coculture with human umbilical vein endothelial cells (HUVECs), miR-378a-5p-transfected MSCs formed a larger number of vascular branches on Matrigel. In the MSCs transfected with miR-378a-5p, the expression of Bcl-2-associated X protein (BAX), which is an important proapoptotic regulator, was decreased, leading to a better survival [93].

It still has to be determined if the proangiogenic effect of miR-378a* in vivo* is confined to tumor angiogenesis, or if this effect is also present in physiological angiogenesis and regenerative neovascularization. Interestingly, new findings in wound healing studies found a rather opposite conclusion. Recently, it was reported that anti-miR-378a-5p enhances wound healing process by upregulating integrin beta-3 and vimentin [116].

The role of the host gene of miR-378a on angiogenesis has also been studied. PGC-1*β* was reported to have opposite effects in ischemia-induced angiogenesis. It was reported that PGC-1*β* induces angiogenesis in skeletal muscle, enhancing the expression of VEGF both* in vitro* and* in vivo* after (transgenic) overexpression [117]. Accordingly, it was also found that VEGFA is upregulated in C2C12 myoblast cell line with PGC-1*β* overexpression. However, after a PCR-based gene array of 84 known angiogenic factors and further RT-PCR of individual genes, they concluded that PGC-1*β* triggered an antiangiogenic program [118]. After inducing hind limb ischemia in PGC-1*β* overexpressing mice, an impaired reperfusion was noticed when compared to wild type littermates [118].

## 7. miR-378a in Inflammation 

The role of inflammation in angiogenesis is studied the most in the context of cancer (e.g., reviewed in [119, 120]) but is certainly not limited to this pathology. Both lymphoid (reviewed in [121, 122]) and myeloid (reviewed in [123]) derived inflammatory cells affect angiogenesis in a stimulating or inhibitory manner. The role of miR-378a in inflammatory cells was reported and its anti-inflammatory effect could be suggested.

NK cells exert potent cytotoxic effects when activated by type I IFN from the host once infected [124]. miR-378a was found to be downregulated in activated NK cells and further proved to target granzyme B. Thus, IFN-*α* activation decreases miR-378a expression and in turn augments NK cell cytotoxicity [124]. Accordingly, suppression of miR-378 targeting granzyme B in NK cells resulted in inhibition of Dengue virus replication* in vivo *[125].

Macrophages are known to play either inhibitory or stimulatory roles in angiogenesis (reviewed by [126]). miRNAs have been proposed to regulate activation and polarization of macrophages (reviewed by [127, 128]). In a study of Rückerl et al. miR-378a-3p was identified as a part of the IL-4-driven activation program of anti-inflammatory macrophages (M2) [129]. miR-378a-3p was highly upregulated after stimulation with IL-4 of peritoneal exudate cells of mice injected with the parasite* Brugia malayi* compared to controls and infected IL-4-knockout mice. The study identified several targets for miR-378a-3p within the PI3 K/Akt signaling pathway, which are important for proliferation but only partially responsible for M2 phenotype [129]. Another study found miR-378a (strand not specified) expression upregulated after stimulation with cytokines like, for example, TNF-*α* and IL-6 [130].

In line with its potential role in macrophages, miR-378a has been suggested as being of importance in the osteoclastogenesis [131]. Mmu-miR-378a (strand not specified) has been found to be upregulated during osteoclastogenesis* in vitro *[131]. Furthermore, serum levels of miR-378a-3p have been shown to correlate with bone metastasis burden in mice injected with mouse mammary tumor cell lines 4T1 and 4T1.2 [132].

## 8. Conclusions 

A growing body of evidence suggests a role for miR-378a as a mediator controlling reciprocally dependent processes in metabolism, muscle differentiation/regeneration, and angiogenesis.

As miR-378a was found to be differentially regulated in different types of cancers and its level is changed in serum of prostate, renal, and gastric cancer patients, it can be considered as a biomarker for those diseases. The correlation between miR-378a expression and disease progression in lung cancer, liver cancer, and rhabdomyosarcoma suggests a further role of this microRNA as a prognostic marker.

Currently, miR-378a is not utilized as a therapeutic molecule. However, if more research will be done to the mechanisms of action, possibilities for therapeutic use of miR-378a could be sought in the field of metabolic disorders, obesity, or tumors. More studies on the effect of miR-378a expression in muscle disorders would also be desirable.

The proangiogenic effect of miR-378a was observed in tumors; however, no studies have been performed on the angiogenic effects of miR-378a in physiological settings or diseases where angiogenesis plays important roles, such as diabetes and cardiovascular diseases. More study has to be done to assess the mechanisms of miR-378a function in blood vessel formation. Of note, in contrast with proangiogenic role of miR-378a, inhibition of miR-378a-5p enhanced wound healing process. This might suggest a role for miR-378a-5p in diseases such as diabetes or in decubitus ulcers, in which wound healing is impaired.

Of note is the confusion that has arises because of a disarray in nomenclature with studies describing the same molecule, miR-378a, as miR-422b, miR-378, or miR-378^*∗*^. In addition, it is not always clear which of the two mature strands of miR-378a is studied. This could lead to misunderstandings and errors in interpreting the data published so far.

## Figures and Tables

**Table 1:** Classification of miR-378 variants. Source: miRBase, version 21, September 2015 [28]. The seed sequence (defined as nucleotides 2–8 from the miRNA 5′-end of the mature miRNA) is in bold font.

Name	Mature strand	Previous ID	Sequence	Location	Host gene
Human					
hsa-miR-378a	hsa-miR-378a-5p	miR-378^*∗*^	5′-c**uccugac**uccagguccugugu-3′	chr5: 149732825–149732890	*PPARGC1B*
				
	hsa-miR-378a-3p	miR-422b	5′-a**cuggacu**uggagucagaaggc-3′	chr5: 149732825–149732890	*PPARGC1B*
		miR-378			
hsa-miR-378b	hsa-miR-378b		5′-a**cuggacu**uggaggcagaa-3′	chr3: 10330229–10330285	*ATP2B2*
hsa-miR-378c	hsa-miR-378c		5′-a**cuggacu**uggagucagaagagugg-3′	chr10: 130962588–130962668	—
hsa-miR-378d-1	hsa-miR-378d		5′-a**cuggacu**uggagucagaaa-3′	chr4: 5923275–5923328	—
hsa-miR-378d-2	hsa-miR-378d		5′-a**cuggacu**uggagucagaaa-3′	chr8: 93916022–93916119	*PDP1*
hsa-miR-378e	hsa-miR-378e		5′-a**cuggacu**uggagucagga-3′	chr5: 170028488–170028566	*DOCK2*
hsa-miR-378f	hsa-miR-378f		5′-a**cuggacu**uggagccagaag -3′	chr1: 23929070–23929147	—
hsa-miR-378g	hsa-miR-378g		5′-a**cugggcu**uggagucagaag-3′	chr1: 94745860–94745900	*LINC01057*
hsa-miR-378h	hsa-miR-378h		5′-a**cuggacu**uggugucagaugg-3′	chr5: 154829458–154829540	*FAXDC2*
hsa-miR-378i	hsa-miR-378i		5′-a**cuggacu**aggagucagaagg-3′	chr22: 41923222–41923297	*TNFRSF13C*
hsa-miR-378j	hsa-miR-378j		5′-a**cuggauu**uggagccagaa-3′	chr17: 37614931–37615039	*DDX52*

Murine					
mmu-miR-378a	mmu-miR-378a-5p	miR-378^*∗*^	5′-c**uccugac**uccagguccugugu-3′	chr18: 61397835–61397900	*PPARGC1B*
	mmu-miR-378a-3p	miR-378	5′-a**cuggacu**uggagucagaagg-3′	chr18: 61397835–61397900	*PPARGC1B*
mmu-miR-378b	mmu-miR-378b		5′-c**uggacuu**ggagucagaaga-3′	chr11: 88352773–88352864	*MSI2*
mmu-miR-378c	mmu-miR-378c		5′-a**cuggacu**uggagucagaagc-3′	chr14: 46954830–46954928	*SAMD4*
mmu-miR-378d	mmu-miR-378d		5′-a**cuggccu**uggagucagaaggu-3′	chr10: 126710282–126710391	—

The “**∗**” sign refers to a nucleotide position not present in the murine and rat miR-378a-3p mature sequence, which is present in the mature human sequence.

**Table 2:** miR-378a is highly conserved between species. Source: miRBase, version 21, September 2015 [28]. The seed sequence (defined as nucleotides 2–8 from the miRNA 5′-end of the mature miRNA) is in bold font.

Species	Name	Sequence
Human	hsa-miR-378a-5p	5′-c**uccugac**uccagguccugugu-3′
	hsa-miR-378a-3p	5′-a**cuggacu**uggagucagaaggc-3′

Mouse	mmu-miR-378a-5p	5′-c**uccugac**uccagguccugugu-3′
	mmu-miR-378a-3p	5′-a**cuggacu**uggagucagaagg•-3′

Rat	rno-miR-378a-5p	5′-c**uccugac**uccagguccugugu-3′
	rno-miR-378a-3p	5′-a**cuggacu**uggagucagaagg•-3′

Pig	ssc-miR-378-1	5′-a**cuggacu**uggagucagaaggc-3′

Cow	bta-miR-378-1	5′-a**cuggacu**uggagucagaaggc-3′

Thirteen-lined ground squirrel	itr-miR-378a	5′-a**cuggacu**uggagucagaaggc-3′

**Table 3:** The known interactions of miR-378a.

miR-378a-3p		miR-378a-5p		Both/unspecified	
Target	Function	Target	Function	Target	Function
*Metabolism*					
*NRF1* [42]^*∗*^	Critical regulator of the mitochondrial function	*CYP2E1* [41]^*∗*^	Involved in conversion of acetyl-CoA to glucose	*cEBPα* [27]^#^	Transcription factor, promotes expression of leptin
*TPM2* [44]^*∗*^	Involved in the regulation of ATPase activity	*ERRγ* [5]^*∗*^	Involved in control of oxidative metabolism	*cEBPβ* [27]^#^	Transcription factor, regulation of genes involved in immune and inflammatory responses
*IGF1R* [57]^#^	Tyrosine kinase receptor, mediates the effects of IGF-1	*GABPα* [5]^*∗*^	Involved in control of oxidative metabolism, nuclear control of mitochondrial function		
*RORA* [69]^#^	Orphan receptor, possibly involved in circadian rhythm	*GDP* [67]^*∗*^	Enzyme involved in GDP-mannose production		
*LDHA* [44]^*∗*^	Involved in the lactic acid cycle	*DDAH* [44]^*∗*^	Involved in the synthesis of arginine from citrulline		
*CRAT* [6]^#^	Catalyzes exchange of acyl groups between carnitine and coenzyme A	*LDHA* [44]^*∗*^	Involved in the lactic acid cycle		
		*MED13* [6]^#^	Component of the mediator complex (transcriptional coactivator)		

*Muscle differentiation and regeneration*					
*MyoR* [7]^#^	Represses MyoD (and thus myogenesis)	*VIM* [44]^*∗*^	Cytoskeletal protein anchoring position of organelles	*Purβ* [69]^#^	Controlling transcription of smooth muscle actin
*ACTN4* [44]^*∗*^	Nonmuscle *α*-actinin isoform, cytoskeletal protein	*Actin* [44]^*∗*^	Cytoskeletal protein, involved in muscle contraction		
*14-3-3-γ* [44]^*∗*^	Regulatory protein highly expressed in muscle	*Hsp70.3* [76]^#^	Involved in cytoprotective responses against stress induced stimuli		
*CASP3* [73]^®^	Involved in activation of apoptosis				
*CTGF* [74]^#^	Connective tissue growth factor				
*TGFβ1* [74]^#^	Involved in cell growth, proliferation, differentiation, and apoptosis				
*IGF1R* [57]^#^	Tyrosine kinase receptor, mediates the effects of IGF-1				
*MAPK *[77, 78]^*®*#^	Involved in proliferation, differentiation cell survival, and apoptosis				

*Angiogenesis*					
*TOB2* [88]^*∗*^	Suppressing cyclin D1	*VEGF-A* [89]^*∗*^	Induction of angiogenesis		
		*SuFu* [8]^*∗*^	Involved in inhibition of SHH pathway		
		*Fus-1* [8]^*∗*^	Involved in RNA binding and tumor suppression		
		*FN1* [112]^#^	Receptor binding integrin		
		*BAX* [93]^*∗*^	Involved in apoptosis, activation of caspases		
		*ITGB3* [116]^#^	Involved in cell adhesion and cell-surface signaling		
		*VIM* [116]^#^	Involved in anchoring position of organelles		

Studies were performed in human (*∗*), mouse (#), or rat (®).
